# Rapid Evaluation of Integral Quality and Safety of Surface and Waste Waters by a Multisensor System (Electronic Tongue)

**DOI:** 10.3390/s19092019

**Published:** 2019-04-29

**Authors:** Evgeny Legin, Olesya Zadorozhnaya, Maria Khaydukova, Dmitry Kirsanov, Vladimir Rybakin, Anatoly Zagrebin, Natalia Ignatyeva, Julia Ashina, Subrata Sarkar, Subhankar Mukherjee, Nabarun Bhattacharyya, Rajib Bandyopadhyay, Andrey Legin

**Affiliations:** 1 Laboratory of Artificial Sensory Systems, ITMO University, Kronverkskiy pr, 49, St. Petersburg 197101, Russia; eugene.legin@gmail.com (E.L.); d.kirsanov@gmail.com (D.K.); ashina.julia91@gmail.com (J.A.); nabarun.bhattacharya@cdac.in (N.B.); bandyopadhyay.rajib@gmail.com (R.B.); 2 Institute of Chemistry, St. Petersburg State University, Mendeleev Center, Universitetskaya nab. 7/9, St. Petersburg 199034, Russia; lesok91@mail.ru; 3 Sensor Systems LLC, pr. Pyatiletok, 2, St. Petersburg 193318, Russia; 4 Institute of Limnology, Russian Academy of Sciences, ul. Sevast’yanova 9, St.-Petersburg 196105, Russia; v.n.rybakin@gmail.com (V.R.); zigzag.56@mail.ru (A.Z.); natali_ignatieva@mail.ru (N.I.); 5 Centre for Development of Advanced Computing (C-DAC), E-2/1, Block-GP, Sector–V, Salt Lake, Kolkata 700091, West Bengal, India; subrata.sarkar@cdac.in (S.S.); subhankar.mukherjee@cdac.in (S.M.); 6 Department of Instrumentation and Electronics Engineering, Jadavpur University, Salt Lake Campus, Plot No.8, Salt Lake Bypass, LB Block, Sector III, Salt Lake City, Kolkata 700098, West Bengal, India

**Keywords:** multisensor system, natural and waste water safety evaluation, water toxicity measurements, bioassay technique, cavitation ultrasound treatment

## Abstract

The paper describes a wide-range practical application of the potentiometric multisensor system (MS) (1) for integral safety evaluation of a variety of natural waters at multiple locations, under various climatic conditions and anthropogenic stress and (2) for close to real consistency evaluation of waste water purification processes at urban water treatment plants. In total, 25 natural surface water samples were collected around St. Petersburg (Russia), analyzed as is, and after ultrasonic treatment. Toxicity of the samples was evaluated using bioassay and MS. Relative errors of toxicity assessment with MS in these samples were below 20%. The system was also applied for fast determination of integral water quality using chemical oxygen demand (COD) values in 20 samples of water from river and ponds in Kolkata (India) and performed with an acceptable precision of 20% to 22% in this task. Furthermore, the MS was applied for fast simultaneous evaluation of COD, biochemical oxygen demand, inorganic phosphorous, ammonia, and nitrate nitrogen at two waste water treatment plants (over 320 samples). Reasonable precision (within 25%) of such analysis is acceptable for rapid water safety evaluation and enables fast control of the purification process. MS proved to be a practicable analytical instrument for various real-world tasks related to water safety monitoring.

## 1. Introduction

The quality and availability of water resources are among major global concerns nowadays. Intense development of urban agglomerations causes additional risks of returning contaminated water into the ecosystem and this implies the necessity of continuous water quality monitoring and water treatment prior to its further use. There are various methods of water physical purification, such as sedimentation and filtration [1,2]; chemical cleaning using special reagents, e.g., flocculants and coagulants [3,4]; and biological treatment assuming decomposition of dangerous substances present in the water by microorganisms [5,6]. It is important to note that most widely applied methods require the use of chemicals, which sometimes can lead to additional water pollution and the necessity of further purification [7]. Water treatment processes employed in different developed countries are following practically the same stages mentioned above, e.g., [8].

Water treatment techniques that may improve its final quality are still being researched. One of the prospective water cleaning techniques is based on cavitation ultrasound treatment (UST). UST helps to decrease chemical contamination [9] and allows for water purification from cyanobacteria (also called blue and green algae) [10,11]. This is an extremely attractive feature of UST since blue and green algae may significantly affect the quality of various natural aquatic environments due to toxins’ emissions.

The UST is based on the ultrasonic cavitation phenomenon promoting the collapse of bubbles in the fluid irradiated with the ultrasound [12]. The gas-vapor mixture inside such bubbles is heated up to 8000 to 12,000 K and the collapse process is characterized by a high radial speed of the bubble’s walls, exceeding the speed of sound, and inducing a local pressure of over 109 Pa. The shockwaves produced in such bubbles may also cause high-voltage (tens of millions of Volts) electric phenomena. Different physical and chemical processes, such as acoustic cavitation, intensive mixing, and breaking-up of chemical bonds, may occur as a result of a bubble’s collapse. A number of accompanying processes, such as intensive dispersion effects, producing emulsions, promoted stratification, and coagulation of solid particles suspended in the liquid and selective destruction of living cells, e.g., of microorganisms, must also be mentioned. These processes may help in reducing the degree of pollution and water toxicity.

Obviously, the UST method does not require any chemical reagents, which is an undoubted advantage of the technique. The design of UST equipment is comparatively simple, the efficacy of bacteria inactivation is high, and there are no undesirable disinfection products after purification [13]. On the other side, the UST technology is highly energy consuming and may not always lead to an improvement of water quality in terms of safety.

Other issues closely related to decent water quality maintenance are the problems of adequate analysis of the water. Though now there are a lot of methods applicable to detailed characteristics of various water features, it is rather obvious that the reliable fast analysis of water quality and its environmental safety must be integral. The determination of multiple discrete parameters would surely take time and there are quite a few of those discrete parameters produced by conventional chemical analysis of the water that are suitable for conclusions about its overall quality.

As a matter of fact, some integral parameters of water safety and quality, such as COD (chemical oxygen demand), BOD (biological oxygen demand), and TOC (total organic carbon), have long been widely used in laboratory practice. They can produce valuable information about important cooperative features of the water, such as oxidizable matter or biological load, without going into multiple details and numerous experimental complications. However, determination of, for example, COD assumes the application of wet chemistry methods with doubtful promise for fully automatic and really fast online analysis [14]. Needless to say, such a procedure performed in the laboratory would demand sampling acquisition and handling that also takes time and professional manpower.

Another type of integral water quality evaluation is based on the determination of its integral safety or toxicity using a bioassay technique. This assessment is based on the evaluation of various reactions (such as mobility, fertility, and survival rate) of living organisms in the studied water sample [15,16]. Such analysis is likely more “global” than most common chemical procedures, but is not that simple, unfortunately. The fastest biotests, such as luminescent bacteria, aquarium fishes, or some crustaceans, need to be properly selected and their living and application conditions must be strictly maintained to get a reliable response pertaining to water safety, but not to whatever else. Even such fast tests would often need several dozens of minutes to be completed, while lingering contamination may totally ruin the performance of biological “devices”. Finally, water safety bioassay tests are rather expensive and are not that widely used as one would anticipate.

We have demonstrated earlier [17,18,19,20] that a properly designed multisensor system (“electronic tongue”) may be successfully applied for water safety or toxicity determination in terms of biotests. The idea of this approach is in establishing a mathematical model relating the response of the set of cross-sensitive chemical sensors in the sample with the toxicity of the sample evaluated by some bioassay. Once established and validated, such a model can be further applied to the toxicity evaluation of unknown samples, without using any biota anymore. Furthermore, it has been found that the same multisensor can be reliably calibrated against responses of different test organisms (*Daphnia magna* [17,18,19], *Chlorella vulgaris*, *Paramecium caudatum* [18], and *Vibrio fischeri* [20]) that demonstrate a more universal performance of the multisensor system in water toxicity evaluation compared to any existing bioassay method. The analysis time in these experiments was as fast as 3 min and, if needed, it might be even further shortened to just a few dozens of seconds. Potentiometric analysis layout can be rather simply automatized. Thus, multisensor arrays are a promising analysis technique for fast integral water safety and quality assessment.

The aim of the present study was twofold.

Firstly, we applied a multisensor system for the evaluation of the safety of a wide range of fresh surface waters (rivers, lakes, ponds) over a considerable geographical area, almost 400 × 500 km, of the North-Western Russia to ensure the universal performance of the system irrespective of the type and hydrological conditions of a water source. Water samples in these experiments were also processed by cavitation ultrasound and the multisensor system was utilized already as a kind of a fiducial technique in these studies.

Secondly, multisensor arrays were applied for the assessment of water environmental safety at two different waste water treatment plants of St. Petersburg (Belyi Island and Zelenogorsk). In this part of the work, we also tried to assess the possibility of electronic tongue application to the analysis of integral water characteristics, especially COD. Along with COD, different discrete parameters, including those that are not really typical targets for potentiometric sensors in multicomponent environments, e.g., phosphates, were also measured. It was also important to check the possible influence of different water purification technologies on the performance of the multisensor system.

The ultimate aim of this work was to demonstrate that a multisensor system is a practicable analytical instrument for routine analysis of real-world waters over a wide range of samples and applications.

## 2. Materials and Methods

### 2.1. Water Samples

In total, 25 water samples were provided by the Institute of Limnology of Russian Academy of Sciences located in St. Petersburg. These were surface waters collected in different locations over North-West Russia—St. Petersburg region, Novgorod region, and Karelia.

In total, 20 water samples were acquired by the co-authors from India from 10 different locations along Ganga river and 10 city ponds over the city of Kolkata (India).

In total, 324 samples of waste water at different treatment stages from two different water purification plans were obtained and analyzed by the standard methods with the help of the stuff of GUP “Vodokanal”, St. Petersburg, Russia.

Each sample was acquired in a plastic bottle with a screw cap. Samples were stored in the refrigerator before and between the measurements. Some of the samples were studied both before and after sonication.

### 2.2. Toxicity Evaluation

A traditional method of water toxicity (water safety) evaluation is a bioassay. Small crustaceans, *Daphnia magna*, can be considered as one of the most common test-objects conventionally used in practice. Their death rate in a studied sample compared to that in a reference is a clear indicator of water toxicity. The advantages of *Daphnia* are in the relatively simple cultivation and high sensitivity to contaminants. On the other hand, such a high sensitivity turns into a disadvantage and may increase the false positive rate of toxicity, e.g., in chlorinated water.

The toxicity of the collected samples was evaluated with the 96-h *Daphnia magna* bioassay. One- or two-day-old *Daphnia magna Straus* were employed. Ten animals were used for a study in each sample and all studies were performed in three replicate runs. In total, 25 mL of a sample was used for each replica. The sample was considered as being toxic when 50% or more of the animals died. The fact of death was determined visually using an optical microscope. The data from three replicas were averaged for further processing.

### 2.3. Ultrasonic Treatment

**Figure 1:**
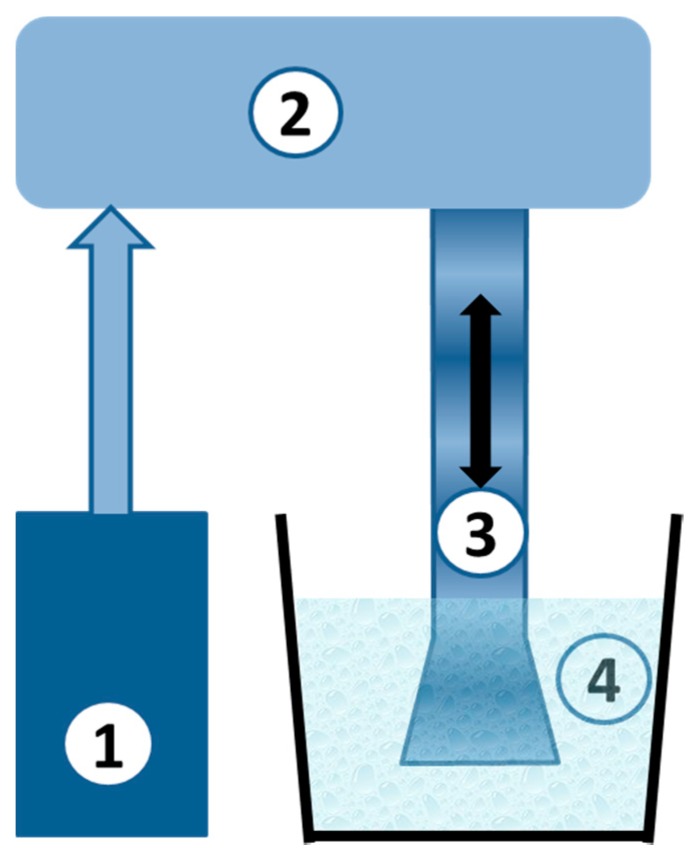
Scheme of the ultrasound device. 1—ultrasound generator, 2—magnetostrictive transducer, 3—waveguide, 4—vessel with water.

Ultrasonic treatment (UST) of water samples was carried out using a homemade (Institute of Limnology of RAS) immersion ultrasonic device (Figure 1), emitting industrially permitted 22 kHz ± 10% resonance working frequency. A low ultrasound frequency promotes the formation of cavitation bubbles, affecting the water quality [21]. The emitter tip (48 mm in diameter) was immersed for five minutes into the water sample (150 mL), which ensured the acoustic pressure ranged from 5.3 to 21 W/cm^2^, thus generating cavitation bubbles. The timing was chosen based on the assumption that the concentration of algae (which may significantly influence the toxicity value) is rapidly reduced after the first five minutes of treatment, and then does not change for a long time [22].

### 2.4. Multisensor Array and Potentiometric Measurements

**Figure 2:**
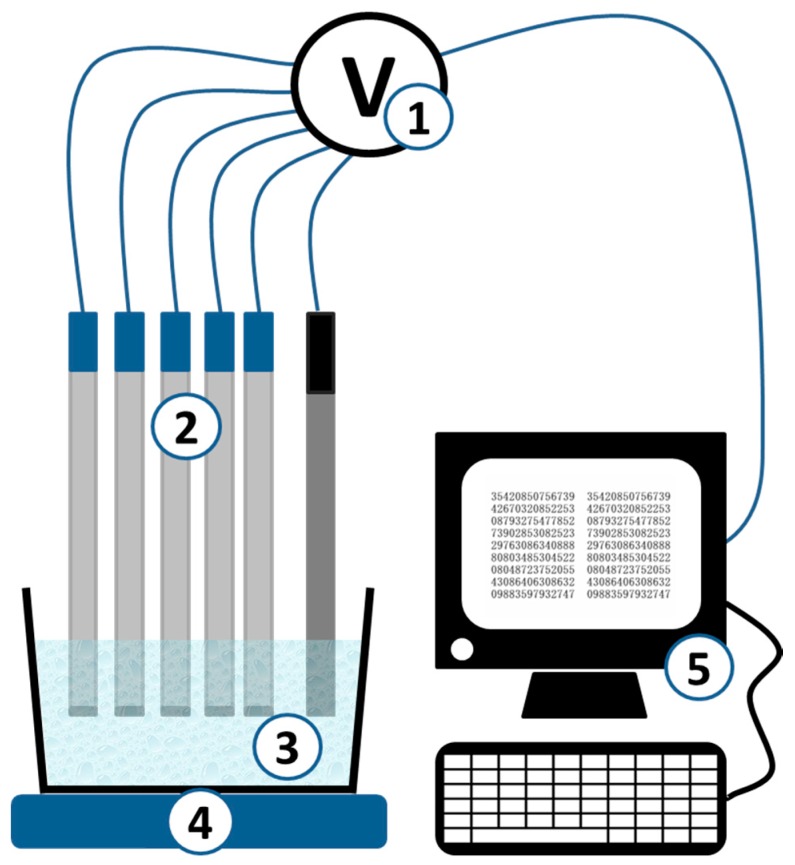
Potentiometric multisensor system. 1—multichannel digital mV-meter, 2—meter, 3—cell with sample, 4—magnet stirrer, 5—personal computer.

The multisensor system applied in this study (Figure 2) was comprised of 20 cross-sensitive potentiometric sensors. Table S1 (Supplementary materials) shows the composition of the sensors. Eight of them were poly(vinyl chloride) (PVC)-plasticized membrane anion-sensitive sensors based on anion-exchangers of various structures, nine were PVC-plasticized membrane cation-sensitive sensors, and three sensors were chalcogenide glass electrodes with a pronounced sensitivity towards heavy metal ions and redox potential. More details on sensor compositions and preparation procedures are available in [23]. All membrane active compounds for PVC-plasticized sensors, i.e., ion-exchangers, plasticizers, and PVC, were of Selectophore^®^ grade from Sigma-Aldrich (Munich, Germany). All components for the synthesis of chalcogenide glasses were of the highest available purity also from Sigma-Aldrich.

The sensors of the array possess sensitivity towards various toxic substances: Heavy metals, organic acids, phenolic substances, etc. The working principles of potentiometric sensors’ response formation are well studied and explained in the literature [24]. The sensors did not exhibit sharp selectivity towards particular ions, but demonstrated high cross-sensitivity to a variety of analytes instead. Each sensor of the array has an individual sensitivity pattern and the number of such sensors being combined together yields a kind of “unresolved spectrum” of a sample. This response can be processed by modern chemometric techniques (the application of mathematical and statistical methods for chemical data analysis) in order to extract qualitative and quantitative information about the analyzed media.

The potentiometric measurements were performed against a standard Ag/AgCl reference electrode in the following galvanic cell:

**Ag|AgCl, KCl_(sat)_|sample solution |sensor membrane |solid contact |AgCl|Ag**

The sensor’s array was connected to the 20-channel digital high input impedance mV-meter (Sensor Systems LLC, St. Petersburg, Russia). Electromotive force (EMF) readings were registered with a 0.1 mV precision and stored in a PC.

The water samples were transferred into the cell manually, however, this procedure can be easily automated and sensors can be placed directly into the water flow. Another important benefit of the proposed methodology is that it does not require sample pretreatments, such as filtration or digestion, due to the employment of potentiometric sensors in the multisensor system. This type of measuring platform tolerates the presence of suspended matter and turbidity in the samples. The samples were measured as is without any treatment under stirring conditions in a 50 mL Teflon cell. The measurement time in each sample was 3 min. The sensor array was washed with three portions of distilled water between the measurements, and the total time of washing was about seven minutes. The reported procedure was sufficient to provide for ±3 mV reproducibility of the sensor readings in the replicated measurements. At least three repetitions were carried out with the potentiometric multisensor system for each sample and these results were averaged for further data processing.

### 2.5. Standard Laboratory Analysis of the Water

The results produced by the multisensor systems on COD, BOD, ammonia nitrogen, nitrate nitrogen, and phosphates were compared to the reference data obtained by the standard certified procedures. These procedures can be performed only in the offline mode, since most of them require sample preparation stages, the employment of chemicals, or a bioassay. These procedures were performed totally independently of the authors of the present paper by the staff of “Vodokanal” laboratory, St. Petersburg, for the samples obtained in Russia and by Vivekananda Institute of Biotechnology, Nimpith Ashram, West Bengal for the samples collected in India.

### 2.6. Data Processing

In order to relate a set of independent variables (sensor responses in our case) with the dependent value (toxicity of the sample), partial least squares (PLS) regression was employed in this study. PLS regression is one of the most widely applied methods in chemometrics. It is based on the latent variable approach and deals with derivative variables constructed as linear combinations of the original variable set. More details on the mathematics behind the method can be found elsewhere [25]. Once the regression model is established, it can be further employed for the prediction of dependent values from the sensor array response without using the reference method. PLS models were computed with The Unscrambler^®^ 9.7 (CAMO Software AS, Oslo, Norway). Evaluation of the PLS model’s performance was conducted using two strategies: Full cross-validation and segmented cross-validation. In full cross-validation, N models were generated, where N is the number of samples, using N–1 samples for training and the remaining one for validation, so that there were in total N samples for validation, but each one for a different model. In segmented cross-validation, 1/3 of the samples was randomly extracted and used as a test set and the remaining 2/3 of the samples were used for constructing a calibration model. This procedure was repeated several times and each time the standard error of cross-validation (RMSECV) was calculated:

(1) $$\mathrm{RMSECV}=\sqrt{\frac{\sum_{i}(y_{i}^{pred}-y_{i}^{real}{)}^{2}}{m}},$$

where *m* is the number of samples in the validation set, $y_{i}^{pred}$ is the toxicity value predicted by the model, $y_{i}^{real}$ is the reference value established with the bioassay. The RMSECV value is a measure of the predictive power of the regression model and it is expressed in the same units as the target parameter of calibration.

Other important parameters of calibration models are the slope, the offset, and the squared correlation coefficient, R^2^, of the “measured vs. predicted” plot. The slope and the squared correlation coefficient describe how well the points of calibration and validation fit. The closer these values to unity, the better the model describes the data. The offset describes the shift of a straight line from the origin of the coordinates, and for robust calibration, the model should be as close to zero as possible.

## 3. Results and Discussion

The first experiment carried out in Russia was related to the analysis of natural waters from rivers, lakes, and ponds over the North-Western of Russia. In total, 25 samples were used for laboratory analysis and measurements with the multisensor system and model building. All samples were measured as is both before and after UST. The samples and their toxicity, as assessed by the bioassay, are summarized in the Table 1.

PLS modeling was employed to relate the response of the sensor array with the sample toxicity. The parameters of the derived PLS models for these data are presented in Table 2. Since the number of samples was rather limited, the model evaluation was performed using segmented cross-validation.

The values of the slopes in the calibration in both cases are close to 1, as well as R^2^ values—this is an indicator of the fact that there is a strong correlation between the response of the multisensor system and water toxicity in terms of *Daphnia magna*. These values are somewhat lower in the model validation, however, it must be pointed out that the employed mode of the segmented cross-validation brings a lot of disturbance to the model, thus the obtained values should be considered as promising. The RMSE values obtained in the model validation may serve as an estimate of the real predictive performance of the model and they look reasonably good taking into account the fact that we modelled complex reactions of living creatures with a simple array of electrochemical sensors. The validation parameters were slightly better for the models derived for the samples after ultrasonic treatment.

The results of the water toxicity determination by the multisensor system were quite reasonable and were in a good agreement with our previous experience reported earlier [17,18,19,20]. One may conclude that the multisensor system can be used for water toxicity evaluation both before and after UST of natural water samples over the variety of different locations and aqueous sources.

It was observed [10] that UST does not necessarily provide a toxicity reduction in all samples, but sometimes leaves the apparent toxicity unaltered or even increases its bioassay produced value. This observation can likely be associated to the presence of cyanobacteria in natural waters. The main bloom period in the natural water sources occurs in summer and different altering varieties of cyanobacteria producing a wide range of ultrasound decomposed products can be found there. Ultrasound may not eliminate these bacteria completely, but, sometimes, may lead to an increase of their biomass instead. Besides, cyanobacteria may release elevated amount of toxins under ultrasound induced stress. Finally, an increase of the *Daphnia magna* death rate might be observed.

It would be necessary to acquire a bigger and more diverse sample set and to apply UST on a wider scale to understand this problem better.

Peculiar geographical correlations were observed for the described data. It was found that it was possible to correctly measure and predict toxicity mostly for the water sources situated to the south and east of St. Petersburg (Ilmen’ Lake, Onega Lake, Volhov River, and their tributaries). About 90% of the data in the best obtained models belonged to the water sources of these areas. However, only the data on the few rivers coming to Ladoga Lake could have been included into the best models and predicted correctly. Water composition and water toxicity issues related to most of the rivers coming to Ladoga Lake should be further studied.

The next experimental session in Russia was devoted to the application of several identical multisensor systems for the evaluation of waste water safety using both integral and discrete parameters, under industrial conditions at functioning water treatment plants around St. Petersburg.

The first of these sessions was performed at the Zelenogorsk (north-western suburb of St. Petersburg) water purification plant and it was devoted to finding out practical analytical tasks to which a multisensor system could be applied to.

Over 30 samples of the waste water were acquired at two different locations of the water purification plant, aerotanks and active sludge chambers, and analyzed. The samples were studied both before and after UST. Additional water samples produced at a model water treatment device were also measured.

Chemometrics methods were applied to derive COD, ammonium, and nitrate’s nitrogen and phosphorus content out of the multisensor system’s data. Reference data about all these parameters were produced by the standard and certified methods at Vodokanal laboratory.

The results of the measurements with the multisensor system were used for simultaneous quantitative determination of all the above mentioned parameters. The values of COD, BOD, ammonia nitrogen, nitrate nitrogen, and phosphates were obtained from the mathematical model relating the response of the sensor array with the numerical values of the corresponding water quality parameters.

**Figure 3:**
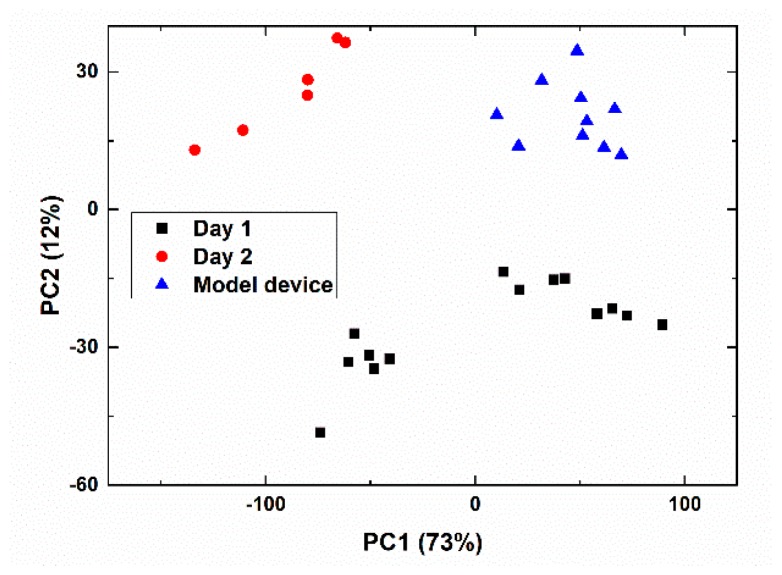
Principal component analysis (PCA) score plot for samples from the Zelenogorsk water treatment plant.

First, we performed sample recognition using principal component analysis (PCA). The loading plot representing sample mapping based on similarities and/or differences of the response of multisensor systems in these samples is shown in Figure 3.

It can be seen in Figure 2 that there is a clear difference between samples taken at different days and times. The samples indexed as Day2 (the second day of the experiment) are clearly separated from Day1 samples (the first day of measurements). We can suppose that the observed time-dependent differences are related to a significant variability of the composition of the waste water coming to the purification plant at different periods of time. Moreover, in the case of Day1, there is a separation between the samples taken at aerotanks (left hand cluster) and active sludge chambers (right hand cluster).

The samples obtained at the model experimental purification device also form a distinct cluster that does not overlap with the others.

Obvious differences in the sample’s integral composition suggest a possibility of establishing a quantitative correlation of the multisensor system’s response with various chemical parameters of the samples.

**Figure 4:**
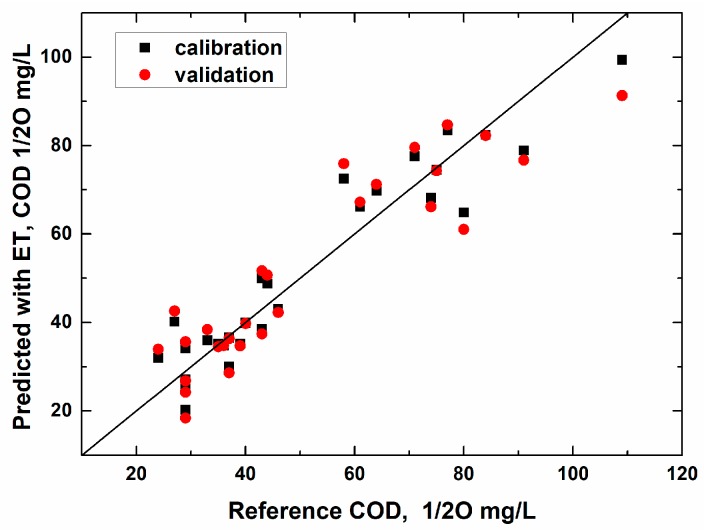
Measured vs. predicted plot of the PLS regression model for chemical oxygen demand (COD) based on three latent variables. Parameters of calibration/validation: slope 0.90/0.85; offset 5.0/7.4; RMSE 7.1/9.1; R^2^ 0.90/0.85.

The PLS algorithm was applied to calculate the values of COD, ammonium, and nitrate nitrogen and phosphorus in the samples. Figure 4 shows such results for COD, while the pictures for the other parameters were pretty similar.

The multisensor system ensured simultaneous determination of all four parameters in a single measurement. The observed correlation coefficients around 0.9 and small number of latent variables (within five variables) confirm the reliability of these results. The precision of the analysis with the multisensor system evaluated by full cross-validation was in the range of 10% to 15% (mean relative error), which is acceptable for express industrial monitoring.

Another piece of this work was devoted to quantitative determination of sodium hypochlorite, which is a very typical reagent for water chlorination and disinfection. However, an elevated amount of hypochlorite may cause adverse effects on human health. Residual hypochlorite in the purified water must be strictly monitored. The multisensor system was applied to the determination of the sodium hypochlorite content in the purified water. Six solutions containing different quantities of sodium hypochlorite ranging from 0.05 to 1.5 mg/L were analyzed with the help of the multisensor system. PLS regression was applied to process the results and the results of cross-validation of the calculated model as a measured-predicted plot are shown in Table 3.

The model developed in this experiment, though somewhat small for a fully reliable prediction, demonstrated the possibility of calculating sodium hypochlorite with an average precision close to ±0.1 mg/L, which shows its applicability to a practically important hypochlorite range.

**Figure 5:**
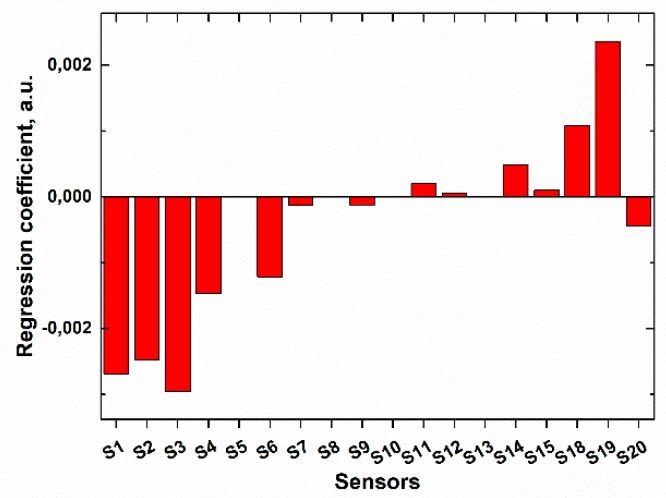
Regression coefficients for different sensors of the array in the PLS model for hypochlorite quantification.

It was proven by calculation of the regression coefficients (Figure 5) that the response of the multisensor system towards sodium hypochlorite was stipulated by the sensitivity to chlorine-containing anions in solution (sensors S1–S6), rather than to sodium (or any other) cations (sensors S7–S15). The redox response of chalcogenide glass sensors (sensors S18–S20) included into the sensor array was also quite informative in this case.

Further tests of the multisensor system were carried out at the waste water purification plant at Belyi Island (western part of St. Petersburg). The overall experimental layout included several stages over this part of the research.

Water samples were acquired from several points of the waste water purification plant, such as the inlet chamber, water tank after mechanical purification, aerotank bulks, aerotank spillways, and bottom active sludge.

The total number of the samples at this stage of this experiment amounted to 180, and 25 of those were later considered as independent blind tests, i.e., they were never used in model building in any way. The reference values of COD were made available (by Vodokanal) for 82 samples, 22 samples for BOD, 112 samples for ammonium and nitrate nitrogen, and for all 155 samples in the case of phosphorus.

It must be noted that though the total number of the samples was significant, the variability of some parameters in those multiple samples from the same site was rather low, which decreased the quality of the prediction models. Since these were real-world samples from a running industrial process it was only possible to increase their quantity, but it was not possible to choose or alter their characteristics for building more reliable models.

Calibration models for each parameter were calculated using different selections of those 155 samples, depending on the availability of the reference data. These models were used for the prediction of parameters in totally unknown, blind samples, an independent test set. The reference values of the parameters for the blind samples were made known for comparison purposes only after all measurement procedures with the multisensor system and related calculations were finalized completely.

The results on the prediction precision of targeted parameters by the multisensor system are shown in Table 4.

It was considered that the models obtained for COD and nitrogen, both ammonia and nitrate, are suitable for semi-quantitative predictions of these parameters. The precision of these predictions might not be of the highest analytical standards, but was suitable for practical purposes—fast, close to real-time monitoring of key water parameters, crucially important for sustainable functioning of a water purification plant.

The models for BOD and phosphates were not highly reliable due to an insufficient number of available samples with reliable reference values (BOD) or low variability of concentrations in the samples in case of phosphorous. However, BOD and phosphorus predictions were still reasonable and could be used for alarm warnings, e.g., to indicate when industrially important threshold values are going to be exceeded.

One should point out some features of phosphorus determination. This element is mostly present in waste water in the chemical form of phosphates. So far, no reliable potentiometric sensors for phosphates were reported, especially those functioning well in the presence of significant amounts of chloride ions, abundant in almost any water, including waste water. This is related to the high hydrophilicity of phosphates hindering their penetration into organic sensor material from aqueous solutions. Active sensor substances ensuring a high affinity to phosphates in the organic phase have not yet been developed. However, the application of adequate multisensor arrays permits phosphorous determination. It can be done due to the cross-sensitivity effects originating in intrinsic correlations between phosphates and other ions causing the sensors to be sensitive in these solutions.

It must be noted that a clear and consistent understanding of the BOD sensing mechanism by potentiometric sensors is lacking, which suggests that the obtained results should be considered as promising, but preliminary.

Relatively low prediction precision in this experiment might be significantly related to a wide dynamic range of some parameters at different stages of water treatment. This can be illustrated by the example of COD. Since the waste water undergoes drastic changes in the purification process, its composition driven properties, including COD, might be dramatically different at the initial and terminating stages of this process. An attempt to simultaneously predict COD values of the water both from the inlet chamber and that at the outcome of the plant, in the framework of a single, unified model, would likely result in elevated prediction errors.

The next experiment was carried out to verify this supposition. As many as 110 new samples (not those described above) were analyzed both by the standard laboratory method and by the multisensor system. In total, 84 of these new samples from the initial purification steps belonged to those with high COD values, while 26 others were the samples of the already purified water with low COD values. Two separate multivariate models were built for these two sample sets and prediction results were evaluated by full cross-validation. The results on the prediction precision of COD by the multisensor system in these two different ranges are shown in Table 5.

Separated modelling of the data ensured dramatic improvement (almost by an order of magnitude, from 69 to 8) of COD prediction precision in purified water samples (total COD range of 9 to 57 mg/L). Furthermore, such separated modelling also decreased the mean relative prediction error of high COD values (230–590 mg/L) by about 25%. The resulting precisions are suitable for quantitative COD prediction with an industrially acceptable precision.

This result clearly proves the necessity of independent consideration of the data acquired by the multisensor system at the initial and final stages of the water purification process. This approach to data handling would perceptibly improve the reliability of all kinds of analysis of the waste water performed using multisensor systems.

**Figure 6:**
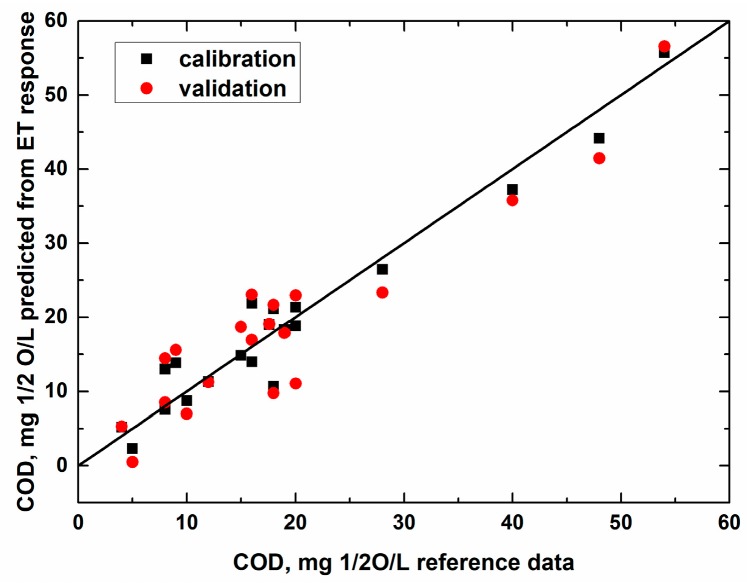
Measured vs. predicted plot of the PLS regression model for COD of natural water samples collected in India. Parameters of calibration/validation: slope 0.94/0.90; offset 1.07/1.61; RMSE 3.1/4.7; R^2^ 0.94/0.87. The model was based on four latent variables.

The experiments with Indian water samples were held jointly by the authors of this paper in Kolkata, India. The plot in Figure 6 shows the measured vs. predicted plot for the PLS regression model intended for COD determination in Ganga River and city pond waters collected around and within Kolkata. In total, 20 samples were analyzed and segmented cross-validation was applied to assess the model performance (six segments, three samples in each).

It can be seen that in the case of the surface waters of India subjected to various types of urban contamination, the COD quantification by means of the potentiometric multisensor system was also possible with reasonable precision.

The authors would like to point out that this result is not at all trivial. Since we are claiming uniform and long-term stable performance of the developed multisensor systems for the applications described above, it was pretty important to demonstrate that closely similar multisensor systems would function uniformly in very different water samples. The range and concentrations of possible agricultural and urban contaminants and their chemical behavior can be significantly different in different countries and different sites and it was important to prove that the multisensor systems are applicable for such diverse tasks.

## 4. Conclusions

The electronic tongue proved to be a practicable analytical instrument for various routine real-world tasks of water safety monitoring.

Multisensor systems were successfully applied for the fast analysis of natural (well over 40 samples) and waste waters (over 320 samples) in urban, rural, and not populated locations over the north-western region of Russia, north-eastern part of India, and at different water treatment plants.

Natural water toxicity was evaluated by the multisensor system in multiple water samples (over 20) from rivers, lakes, and ponds both before and after treatment by cavitation ultrasound (UST). A high correlation of the multisensor results with the water toxicity levels evaluated by *Daphnia magna* was observed for untreated samples (MRE around 20%), in full agreement with previously published results. However, the samples after UST were clearly divided into two clusters, which were correlated to the sample’s geographical origin. For most of the samples, originating from various locations to the south and east of St. Petersburg, a good correlation with the bioassay toxicity was established after UST as well (MRE 20%). However, for water samples collected around Ladoga Lake, the results of UST were found to be inconsistent, which indicates the need for future intensive studies.

Integral water quality based on fast evaluation of COD values was carried out on 20 natural water samples from Ganga River and ponds over the North-Eastern part of India, in the city of Kolkata. The results in this case also showed the reliable performance of the multisensor system, obtaining an MRE of the COD determination within 20% to 25%. This result confirms the applicability of the multisensor system to the analysis of widely variable water samples with different contamination patterns.

Multisensor systems were further applied for the evaluation of integral and discrete parameters of waste water at two water treatment plants around St. Petersburg (Belyi Island and Zelenogorsk). Good correlations (R^2^ 0.85 for cross-validation) of the multisensor results with COD values produced by standard chemical analysis were observed for both locations, confirming the high usefulness of the multisensor systems for integral water quality analysis. Furthermore, a number of discrete water analytical parameters, such as ammonium and nitrate’s nitrogen and phosphorous, could also be determined with encouraging precision (around 25%) using multisensor arrays. It was quite important that the whole realm of integral and discrete characteristics was calculated on the basis of the same set of measurements with the multisensor system, which proves the possibility of obtaining various data on multiple parameters simultaneously, without any additional laboratory procedures, materials, or a significant workload.

It must be pointed out that all analyses with multisensor systems described in this paper can be fully automatized and performed in an unmanned and remote mode.

Thus, a properly designed and trained multisensor system can be seriously considered as a powerful analytical instrument for the various real-world tasks of water quality monitoring.

## Figures and Tables

**Table 1:** Toxicity values of the water samples evaluated by the *Daphia magna* bioassay before and after ultrasonic treatment (UST).

No	The Site (River, Lake, Pond)	Toxicity before UST	Toxicity after UST
1	Pola	10	0
2	Shelon’	30	20
3	Veryasha	10	10
4	Ilmen’ Lake	40	0
5	Volhov-2	80	10
6	Volhov-Novgorod	70	30
7	Tigoda	60	0
8	Perehoda	60	20
9	Nisha	10	10
10	Msta	20	20
11	Park Pobedy pond	100	10
12	Big Pond	0	10
13	Volhov-Kotovitsy	100	0
14	Vytegra	40	20
15	Volhov-Kirishi	50	30
16	Volhov–upper power plant	30	10
17	Volhov–below power plant	10	10
18	Megra	80	40
19	Tuloksa	10	60
20	Mor’e	10	70

**Table 2:** The parameters of the “measured vs. predicted” plots for partial least squares (PLS) regression models predicting water toxicity in terms of *Daphnia magna* (death rate 0–100%) from the multisensor system response.

Experimental Layout/Results	Slope	Offset	RMSE	R^2^
Before ultrasonic treatment (20 samples)				
Calibration	0.95	2.2	7	0.95
Segmented cross-validation (7 segments 3 samples, 7 LV, 88.59% of explained variance in Y)	0.76	9.3	21	0.65
After ultrasonic treatment (20 samples)				
Calibration	0.93	1.2	5	0.93
Segmented cross-validation (7 segments 3 samples, 5 LV, 90.35% of explained variance in Y)	0.83	5.1	11	0.69

**Table 3:** The parameters of “measured vs. predicted” plots for PLS models predicting the sodium hypochlorite content from the multisensor system response.

Experimental Layout/Results	Slope	Offset	RMSE	R^2^
Calibration	0.99	0.00	0.05	0.99
Full cross-validation	0.99	0.01	0.12	0.95

**Table 4:** The values of root mean square error of prediction (RMSEP) for water parameter’s evaluation using the multisensor system.

Parameter	The Range of the Parameter in the Calibration Samples, mg/L	RMSEP, mg/L
COD	22–427	69
Nitrate nitrogen	0.1–9.5	1.7
Ammonia nitrogen	0.3–30.0	3.9
Phosphorous	0.02–44.00	8.4
BOD	2–185	26

**Table 5:** The values of RMSEP for the determination of COD by the multisensor system employing two separate concentration ranges.

Parameter	The Range of the Parameter in the Calibration Samples, mg/L	RMSEP, mg/L
COD at inlet	230–590	52
COD at outlet	9–57	8

## Supplementary Materials

The following are available online at www.mdpi.com/xxx/s1, Table S1: Composition of the sensor array.
